# Dehydroeburicoic Acid from *Antrodia camphorata* Prevents the Diabetic and Dyslipidemic State via Modulation of Glucose Transporter 4, Peroxisome Proliferator-Activated Receptor α Expression and AMP-Activated Protein Kinase Phosphorylation in High-Fat-Fed Mice

**DOI:** 10.3390/ijms17060872

**Published:** 2016-06-03

**Authors:** Yueh-Hsiung Kuo, Cheng-Hsiu Lin, Chun-Ching Shih

**Affiliations:** 1 Department of Chinese Pharmaceutical Sciences and Chinese Medicine Resources, China Medical University, Taichung City 40402, Taiwan; kuoyh@mail.cmu.edu.tw; 2 Department of Biotechnology, Asia University, Taichung City 41354, Taiwan; 3 Department of Internal Medicine, Fengyuan Hospital, Ministry of Health and Welfare, Fengyuan District, Taichung City 42055, Taiwan; keny71@pchome.com.tw; 4 Graduate Institute of Pharmaceutical Science and Technology, College of Health Science, Central Taiwan University of Science and Technology, No. 666 Buzih Road, Beitun District, Taichung City 40601, Taiwan

**Keywords:** dehydroeburicoic acid, *Antrodia camphorata*, high-fat-diet, glucose transporter 4, peroxisome proliferator-activated receptor α, fatty acid synthase

## Abstract

This study investigated the potential effects of dehydroeburicoic acid (TT), a triterpenoid compound from *Antrodia camphorata*, *in vitro* and examined the effects and mechanisms of TT on glucose and lipid homeostasis in high-fat-diet (HFD)-fed mice. The *in vitro* study examined the effects of a MeOH crude extract (CruE) of *A. camphorata* and Antcin K (AnK; the main constituent of fruiting body of this mushroom) on membrane glucose transporter 4 (GLUT4) and phospho-Akt in C2C12 myoblasts cells. The *in vitro* study demonstrated that treatment with CruE, AnK and TT increased the membrane levels of glucose transporter 4 (GLUT4) and phospho-Akt at different concentrations*.* The animal experiments were performed for 12 weeks. Diabetic mice were randomly divided into six groups after 8 weeks of HFD-induction and treated with daily oral gavage doses of TT (at three dose levels), fenofibrate (Feno) (at 0.25 g/kg body weight), metformin (Metf) (at 0.3 g/kg body weight) or vehicle for another 4 weeks while on an HFD diet. HFD-fed mice exhibited increased blood glucose levels. TT treatment dramatically lowered blood glucose levels by 34.2%~43.4%, which was comparable to the antidiabetic agent-Metf (36.5%). TT-treated mice reduced the HFD-induced hyperglycemia, hypertriglyceridemia, hyperinsulinemia, hyperleptinemia, and hypercholesterolemia. Membrane levels of GLUT4 were significantly higher in CruE-treated groups *in vitro.* Skeletal muscle membrane levels of GLUT4 were significantly higher in TT-treated mice. These groups of mice also displayed lower mRNA levels of glucose-6-phosphatase (G6 Pase), an inhibitor of hepatic glucose production. The combination of these agents produced a net hypoglycemic effect in TT-treated mice. TT treatment enhanced the expressions of hepatic and skeletal muscle AMP-activated protein kinase (AMPK) phosphorylation in mice. TT-treated mice exhibited enhanced expression of hepatic fatty acid oxidation enzymes, including peroxisome proliferator-activated receptor α (PPARα) and increased mRNA levels of carnitine palmitoyl transferase Ia (CPT-1a). These mice also exhibited decreased expression levels of lipogenic fatty acid synthase (FAS) in liver and adipose tissue and reduced mRNA levels of hepatic adipocyte fatty acid binding protein 2 (aP2) and glycerol-3-phosphate acyltransferase (GPAT). These alterations resulted in a reduction in fat stores within the liver and lower triglyceride levels in blood. Our results demonstrate that TT is an excellent therapeutic approach for the treatment of type 2 diabetes and hypertriglyceridemia.

## 1. Introduction

Diabetes mellitus is the core of multiple etiologies of chronic hyperglycemia due to faults in insulin secretion/action or both. Type 2 diabetes affects an estimated 90%–95% of all diabetes cases, and it is characterized by the metabolic defect known as insulin resistance [1]. The pathogenesis of type 2 diabetes includes insulin resistance and 5% of patients with impaired β-cell function. Insulin resistance occurs when insulin fails to exhibit its numerous biological effects, and basic symptomatology includes dyslipidemia, obesity, and hypertension [2]. The progress of insulin resistance is the product of heredity and life style factors. Food is the core determinant that regulates these metabolic disorders. The proportion of fat in the diet may be particularly important.

The pancreas secretes insulin and maintains normal glucose homeostasis, assists glucose uptake, and modulates carbohydrate and lipid metabolism [2]. Glucose transporter 4 (GLUT4) plays an important role in the regulation of blood glucose homeostasis. Insulin stimulates glucose uptake principally via the promotion of GLUT4 translocation from an intracellular location to the membrane. Expression level of membrane GLUT4 is proposed to evaluate the insulin responsive glucose transporter GLUT4 from intracellular storage sites to the plasma membrane in skeletal muscle that occurs faulty in insulin resistance [3]. Akt/PKB signaling plays a central role in insulin stimulated glucose uptake in both muscle and adipose tissue, and the effect of insulin on glucose uptake in peripheral tissue via Akt/PKB is through its ability to translocate GLUTs to the cell membrane, thereby facilitating glucose uptake to stimulate glucose uptake by modulating glucose transporter 4 (GLUT4) [4]. Impaired GLUT4 expression, GLUT4 translocation and/or insulin pathway may lead to insulin resistance and hyperglycemia [5]. Therefore, the therapeutic goal is the improvement of plasma membrane GLUT4 translocation or content.

AMP-activated protein kinase (AMPK) is responsible for modulation of cell and whole body energy metabolism. Evidences suggest that peripheral glucose uptake into skeletal muscle (the main disposal site for glucose) can be promoted via two distinct pathways including insulin-dependent mechanisms resulting in activation of Akt/PKB and contraction-mediated stimulation or hypoxia-mediated stimulation of AMPK [4]. The pathway of AMPK phosphorylation belongs to the different superior modulator of GLUT4 translocation [3]. Insulin resistance causes lipid and glucose catabolism. Thus, AMPK activators are expected to be favorable therapies in the treatment of diabetes and related disorders.

Metformin is a clinical antidiabetic drug for the treatment of type 2 diabetes [4]. Metformin activates AMPK [4]. Metformin is a low-potency compound that is used at high doses but results in only modest net efficacy. Significant side effects can also occur [4,5].

**Figure 1:**
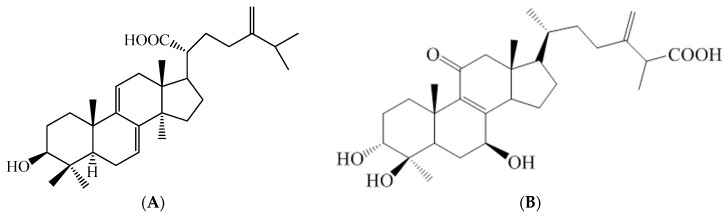
Chemical structures of (**A**) dehydroeburicoic acid (TT) and (**B**) antcin K (AnK).

*Antrodia camphorata* (Polyporaceae, Aphyllophorales) is as a traditional Chinese medicine in Taiwan. *A. camphorata* is a rare and expensive medicine because it only grows on the inner heartwood wall of the endemic evergreen *Cinnamomum kanehirai*. *A. camphorata* exhibits numerous physiological functions, including a hypoglycemic effect [6,7,8]. The fruiting body consists of terpenoids [9], including antcin A, B, C, E, F, K, zhankuic acid A, B, C, D, E, 15α-acetyl-dehydrosulphurenic acid, dehydroeburicoic acid (TT) (Figure 1A), dehydrosulphurenic acid (TR4), methyl antcinate G, H, and eburicoic acid (TR1). Antcin K (AnK) (Figure 1B) is the main constituent of the fruiting body of *A. camphorata*. The identified compounds of mycelium of *A. camphorata* contain antroquinonol and 4-acetylantroquinonol B, succinic and maleic derivatives. Solid culture of the fruiting body and filtrate in submerged cultures exhibit hepatoprotective and antioxidant activities [10,11]. Dehydroeburicoic acid was isolated from *Poria cocos* [9,10] and *A. camphorata* [10,12]. A previous study demonstrated the *in vivo* metabolism of 13 terpenoids in *A. camphorata* using LC/MS/MS in rat plasma after oral administration. Plasma concentrations of ergostanoids were much higher than lanostanoids, and the ergostanoids underwent reduction and hydroxylation reactions *in vivo* [13]. The mean residence time (MRT) ranged from 3 to 6 h. The lanostanoids were not active to metabolic reactions, and they were slowly eliminated with an MRT of 9–16 h [13].

C57BL/6J mice on a high-fat diet (HFD) induces early type 2 diabetes and markedly increases adipose weights, produces resistance to insulin, and increases in blood glucose, total cholesterol (TC), and triglyceride levels. Phosphorylation of threonine 172 (Thr-172) of the α subunit is essential for AMPK activity [14]. Skeletal muscle and adipose tissue play unique roles in the regulation of insulin-dependent glucose homeostasis [15]. Skeletal muscle is likely the primary site of whole-body insulin-mediated glucose uptake [16,17]. Adipose tissue accounts for a small fraction of glucose disposal after a meal, and muscle takes up the majority of glucose [18,19]. Therefore, this study evaluated MeOH crude extract (CruE) from *A. camphorata* and AnK (the main consistent of this mushroom) on expression levels of GLUT4 and phospho-Akt *in vitro*. The antidiabetic and antihyperlipidemic activities and mechanisms of TT were also examined in a HFD-fed mouse model. This mouse model induces type 2 diabetes. The current study investigated whether TT increased membrane glucose uptake transport and AMPK phosphorylation. This study also investigated the peripheral tissues of HFD-induced mice to examine TT-treated effects on targeted genes and the levels of fatty acid oxidation peroxisome proliferator-activated receptor α (PPARα), lipogenic fatty acid synthase (FAS), gluconeogenic glucose 6-phosphoatase (G6 Pase), and carnitine palmitoyl transferase Ia (CPT-1a).

## 2. Results

### 2.1. Expression Levels of Membrane GLUT4 and Akt Phosphorylation in Vitro

**Figure 2:**
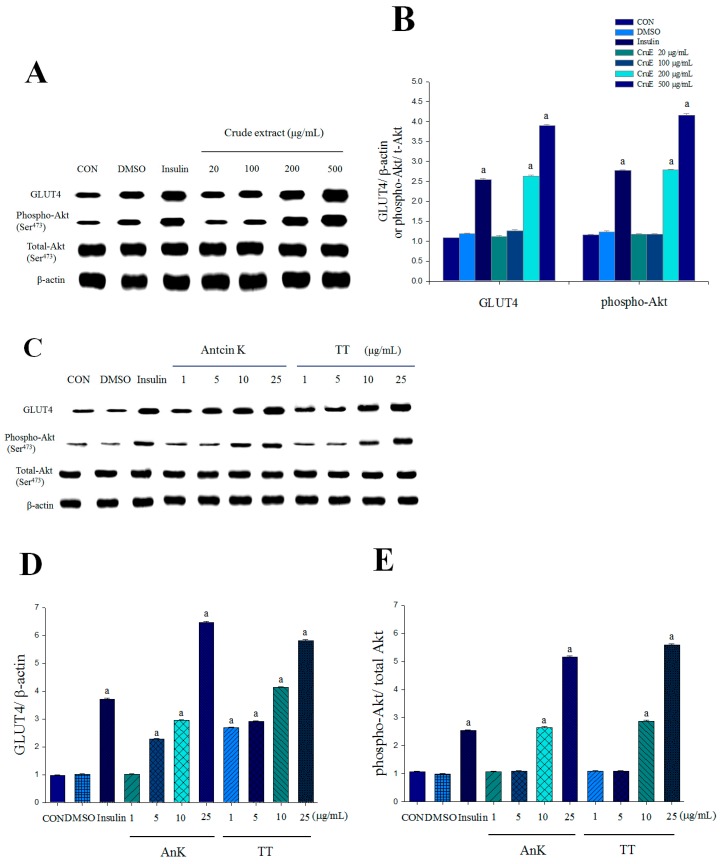
Effects of MeOH crude extract (CruE) and comparative effects of antcin K (AnK) and dehydroeburicoic acid (TT) on membrane glucose transporter 4 (GLUT4) and phospho-Akt/total-Akt *in vitro*: (**A**,**C**) representative blots in C2C12 myoblasts cells; (**B**,**D**,**E**) quantification of the GLUT4 protein contents and the ratio of phospho-Akt to total Akt. C2C12 skeletal myoblasts cells were treated with MeOH crude extract or compounds as described under Material and Methods, and equal amounts of lysates were resolved by SDS-PAGE and blotted for GLUT4, Akt, and phospho-Akt (Ser^473^). All values are means ± SE (*n =* 3). ^a^ *p* < 0.001 compared with the control group.

Treatment with insulin and CruE (200 and 500 μg/mL) increased the expression levels of membrane GLUT4 and phospho-Akt (Ser^473^)/total Akt (Figure 2A,B). Treatment with insulin, AnK (10, and 25 μg/mL), and TT (10 and 25 μg/mL) enhanced the expression levels of phospho-Akt (Ser^473^)/total Akt (Figure 2C,E). TT treatment (between 1 and 25 μg/mL) was not toxic to C2C12 myotubes in the MTT (3-(4,5-dimethylthiazol-2-yl)-2,5-diphenyltetrazolium bromide) assay (data not shown). Treatment with insulin, AnK (5, 10, and 25 μg/mL) and TT (1, 5, 10 and 25 μg/mL) increased the expression levels of membrane GLUT4 (Figure 2C,D).

### 2.2. Metabolic Parameters

Table 1 shows that the HFD-fed mice exhibited increased final body weights and body weight gain compared to the control mice (CON: received low-fat diet plus vehicle) at the end of the experiment. Three groups were treated with HFD and TT at 10, 20, or 40 mg/kg/day body weight (groups TT1, TT2, and TT3, respectively). Three comparative groups were administered HFD and fenofibrate (Feno: 0.25 g/kg/day body weight), metformin (Metf: 0.3 g/kg/day body weight) or distilled water (HF: high-fat control). TT2-, TT3-, Feno-, and Metf-treated mice exhibited decreased final body weight compared to the vehicle-treated HF mice. TT1-, TT2-, TT3-, Feno-, or Metf-treated mice exhibited reduced bodyweight gain. The HF mice consumed less than the CON mice. The food intake of TT3- and Feno-treated mice was lower than the HF mice. The HFD-fed mice exhibited enhanced absolute weights of epididymal, mesenteric, and retroperitoneal white adipose tissue, visceral fat and brown adipose tissue (BAT). TT3-, Feno-, and Metf-treated mice significantly reduced the weights of epididymal and retroperitoneal WAT. TT2-, TT3, Feno-, and Metf-treated mice exhibited decreased the weights of visceral fat and BAT. TT1-, TT2-, TT3-treated mice decreased liver weights, but Metf-treated mice significantly increased liver weights compared to the HF mice (Table 1).

### 2.3. Blood Glucose, Insulin, and Leptin Levels

HFD-induced mice exhibited higher blood glucose, insulin, and leptin levels than the CON mice. TT1-, TT2-, TT3-, Feno-, and Metf-treated mice had significantly lower blood glucose levels. TT1-, TT2-, TT3-, Feno-, and Metf-treated mice exhibited significantly decreased blood insulin and leptin levels, and TT3 treatment did not differ from the CON group (Table 1).

### 2.4. Blood Triglyceride, Total Cholesterol, and Hepatic Lipid

Plasma triglyceride (TG), total cholesterol (TC), and free fatty acid (FFA) were higher in the HF group compared to the CON group. TT1, TT2, TT3, Feno, and Metf treatment reduced plasma TG, TC, and FFA levels (Table 1). Feeding an HFD increased the levels of hepatic total lipids and triacylglycerol, and TT1, TT2, TT3, Feno, and Metf treatment reduced the levels of hepatic total lipids and triacylglycerol compared to the HF group (Table 1).

### 2.5. Pathological Examination

**Figure 3:**
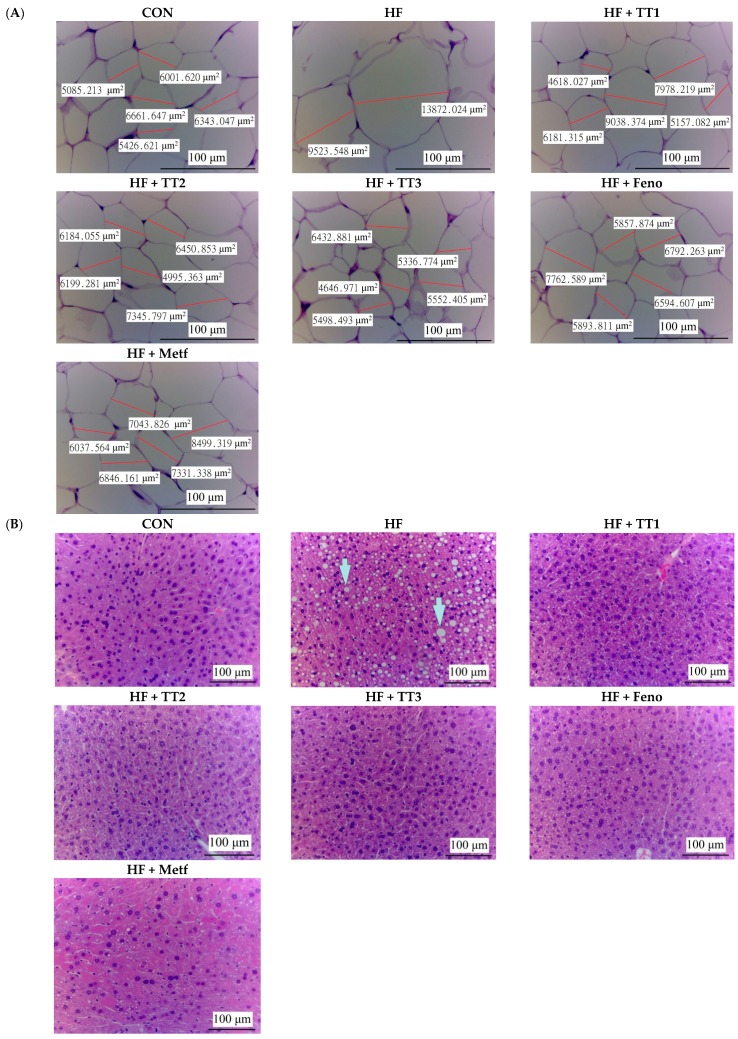
Pathological effects of dehydroeburicoic acid (TT) on (**A**) epididymal white adipose tissue (WAT); and (**B**) liver tissue of mice in the control (CON), high-fat-diet plus vehicle (distilled water) (HF), HF+ TT1, HF + TT2, HF + TT3, HF + Fenofibrate (Feno), or HF + metformin (Metf) groups by hematoxylin and eosin-staining. Magnification: 10 (ocular) × 20 (object lens). TT1, TT2, and TT3: dehydroeburicoic acid (TT1: 10, TT2: 20, and TT3: 40 mg/kg bodyweight); Feno: fenofibrate (250 mg/kg body weight), Metf: metformin (300 mg/kg body weight).

HFD-induction resulted in adipocyte hypertrophy (the average areas of the adipocytes in the HF group and the CON group were 11,212.6 ± 485.2 and 6033.1 ± 258.8 μm^2^, respectively). Treatment with TT1, TT2, TT3, Feno, and Metf caused adipocyte atrophy compared to the HF group. The average areas of the adipocytes in the TT1, TT2, TT3, Feno, Metf groups were 6872.5 ± 160.8 μm^2^, 6530.2 ± 148.2 μm^2^, 5972.4 ± 279.5 μm^2^, 6270.5 ± 165.4 μm^2^, and 6919.5 ± 195.1 μm^2^, respectively (Figure 3A). HFD induction caused obvious hepatic ballooning degeneration because of glycogen accumulation in the nucleus. It was observed that HFD caused a significant ballooning degeneration in the liver of HF mice. This was associated with hepatocyte death and glycogen accumulation in the center, and the nucleolus was squeezed into the other side, implying that the ballooning (the indicated arrow). However, hepatocytes displayed no ballooning after treatment with TT1, TT2, TT3, Feno, and Metf (Figure 3B).

### 2.6. mRNA Levels of Targeted Hepatic Genes

**Figure 4:**
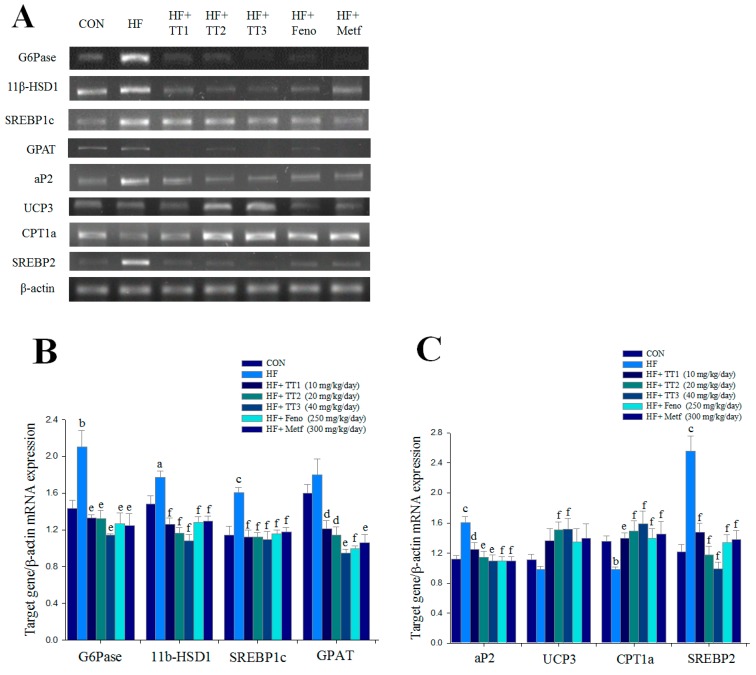
Semiquantative reverse transcription-polymerase chain reaction (RT-PCR) analysis on G6 Pase, 11β hydroxysteroid dehydrogenase (11β-HSD1), sterol response element binding protein-1c (SREBP1c), glycerol-3-phosphate acyltransferase (GPAT), adipocyte fatty acid binding protein (aP2), uncoupling protein 3 (UCP3), carnitine palmitoyl transferase Ia (CPT1a), and SREBP2 mRNA levels in liver tissue of the mice by oral gavage dehydroeburicoic acid (TT1, TT2, and TT3, 10, 20 and 40 mg/kg body weight), or fenofibrate (Feno; 250 mg/kg body weight), or metformin (Metf; 300 mg/kg body weight): (**A**) representative image; (**B**,**C**) quantification of the ratio of target gene to GAPDH mRNA expression. Total RNA (1 μg) isolated from tissue was reverse transcripted by MMLV-RT; 10 μL of RT products were used as templates for PCR. The expression levels of G6 Pase, 11β-HSD1, SREBP1c, GPAT, aP2, UCP3, CPT1a, and SREBP2 mRNA were measured and quantified by image analysis. Values were normalized to β-actin mRNA expression. All values are means ± SE (*n* = 9). ^a^ *p* < 0.05, ^b^ *p* < 0.01, and ^c^ *p* < 0.001 compared with the control (CON) group; ^d^ *p* < 0.05, ^e^ *p* < 0.01, and ^f^ *p* < 0.001 compared with the high-fat-diet plus vehicle (distilled water) (HF) group.

HFD elicited higher mRNA levels of G6Pase, 11β-HSD1, sterol response element binding protein-1c (SREBP-1c), adipocyte fatty acid binding protein (aP2), and SREBP2, but lower in mRNA levels of carnitine palmitoyl transferase Ia (CPT-1a). TT1, TT2, TT3, Feno, and Metf treatments reduced the mRNA levels of G6Pase, 11β-HSD1, SREBP1c, glycerol-3-phosphate acyltransferase (GPAT), and aP2 and enhanced mRNA levels of CPT-1a. TT2 and TT3 treatments increased the mRNA levels of uncoupling protein 3 (UCP3) (Figure 4A–C).

### 2.7. Targeted Gene Expression Levels in Different Tissues

**Figure 5:**
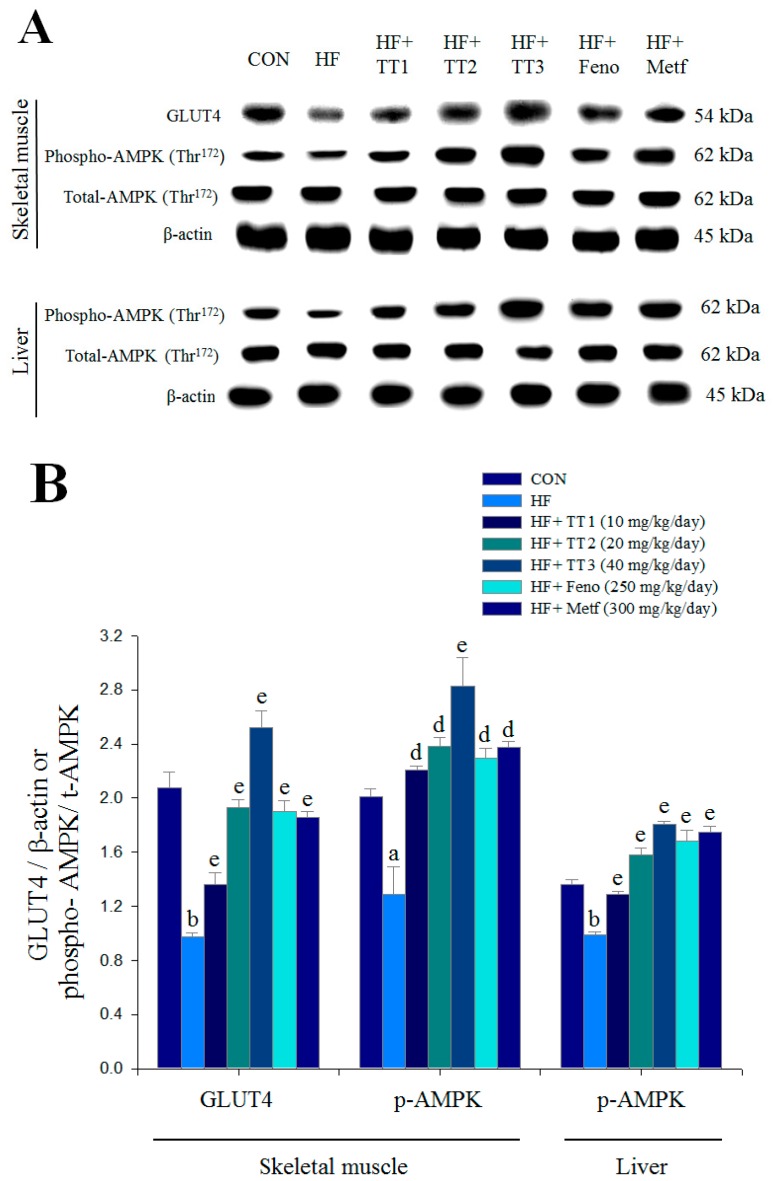
The skeletal muscular expression levels of membrane GLUT4 and phospho-AMP-activated protein kinase (AMPK) (Thr^172^), and hepatic expression levels of phospho-AMPK (Thr^172^) from the high-fat diet (HFD)-fed mice by oral gavage dehydroeburicoic acid (TT): (**A**) representative image; (**B**) quantification of the GLUT4 expression levels and the ratio of phospho-AMPK to total AMPK (mean ± SE, *n* = 9). Protein was separated by 12% SDS-PAGE detected by Western blot. ^a^ *p* < 0.05 and ^b^ *p* < 0.01 compared with the control (CON) group; ^d^ *p* < 0.01, and ^e^ *p* < 0.001 compared with the high-fat-diet plus vehicle (distilled water) (HF) group. TT, dehydroeburicoic acid (TT1, TT2, and TT3, 10, 20, and 40 mg/kg body weight, respectively); fenofibrate (Feno, 250 mg/kg body weight), or metformin (Metf, 300 mg/kg body weight).

The HFD-induced mice exhibited lower expression levels of muscular membrane GLUT4, and this expression was significantly enhanced by TT1, TT2, TT3, Feno, and Metf treatment. HFD-induced mice exhibited decreased hepatic and skeletal muscle expression levels of phospho-AMPK/total AMPK, and these reductions were significantly reversed in TT1-, TT2-, TT3-, Feno-, and Metf-treated mice (Figure 5A,B).

**Figure 6:**
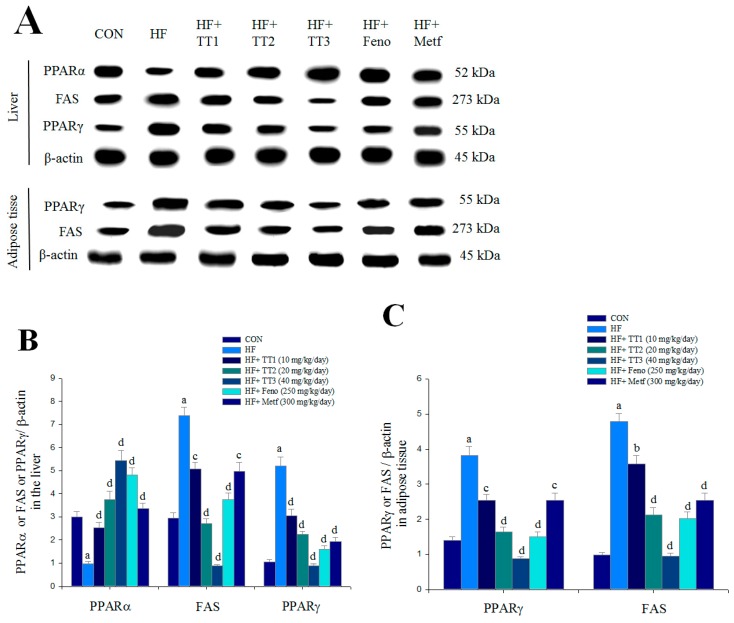
The hepatic expression levels of peroxisome proliferator-activated receptor α (PPARα), FAS, and PPARγ, and the adipose expression levels of fatty acid synthase (FAS) and PPARγ from the HFD-fed mice by oral gavage dehydroeburicoic acid (TT): (**A**) representative image; (**B**,**C**) quantification of the expression levels of PPARα, FAS, and PPARγ in the liver and expression levels of FAS and PPARγ in adipose tissue. Protein was separated by 12% SDS-PAGE detected by Western blot. All values are means ± SE (*n* = 9). ^a^ *p* < 0.001 compared with the control (CON) group; ^b^ *p* < 0.05, ^c^ *p* < 0.01, and ^d^ *p* < 0.001 compared with the high-fat-diet plus vehicle (distilled water) (HF) group. TT, dehydroeburicoic acid (TT1, TT2, and TT3, 10, 20, and 40 mg/kg body weight, respectively); fenofibrate (Feno, 250 mg/kg body weight), or metformin (Metf, 300 mg/kg body weight).

The HFD-induced mice had reduced hepatic expression levels of PPARα, and this was significantly enhanced by TT1, TT2, TT3, Feno, and Metf treatment. The HFD-induced mice had significantly enhanced hepatic and adipose expression levels of FAS and PPARγ. Hepatic and adipose expression levels of FAS and PPARγ were significantly reduced in the TT1-, TT2-, TT3-, Feno-, and Metf-treated groups (Figure 6A–C).

## 3. Discussion

The present study performed CruE (crude extract) experiments in a cell model, and one of the substances isolated from *A. camphorata*, TT, was investigated in an animal model. TT exhibited excellent effects on glucose-lowering and circulating lipids in STZ-induced mice in our recent study [8]. Metformin is a widely used antidiabetic drug for the treatment of type 2 diabetes [4]. Metformin is a low-potency compound that is used at high doses, which results in only modest net efficacy and significant side effects [4,5]. Therefore, an understanding of the effects of TT and its molecular basis of action in HFD-fed mice is an important focus of research toward improved therapeutic approaches to type 2 diabetes and related disorders.

The HFD-fed mice model induces insulin resistance, which represents over 90% of diabetes cases. This study compared two groups treated with the clinical hypolipidemic drug Feno or the antidiabetic drug Metf. Feno also exhibits good glycemic control [20,21]. Our results demonstrated that HFD-induced mice developed hyperglycemia, hypertriglyceridemia, hypercholesterolemia, and hyperinsulinemia. TT treatment lowered blood glucose, triglyceride, total cholesterol, and insulin levels, ameliorated hyperglycemia and hyperlipidemia, and exhibited protection against HFD-induced insulin resistance.

One novel finding of this study is that TT treatment strikingly lowered blood glucose levels by 34.2%, 39.7%, or 43.4%. The antihyperglycemic activity of TT at 10 mg/kg/day was 34.2%, and the glucose-lowering effects of TT at 20 and 40 mg/kg/day were more effective than Feno (37.0%) or Metf (36.5%). These findings indicate that TT exhibited an excellent antidiabetic and antihyperlipidemic effects in HFD-fed mice.

This study first investigated the expression levels of membrane GLUT4 following TT treatment because GLUT4 plays a key role in blood glucose homeostasis [22]. The expression level of membrane GLUT4 is used to evaluate the insulin responsiveness of GLUT4 from intracellular storage sites to the plasma membrane in skeletal muscle, which is faulty in insulin resistance [22]. *In vitro* treatment with insulin and CruE (at 200 and 500 μg/mL) significantly increased the expression levels of membrane GLUT4 and phospho-Akt in C2C12 myoblast cells. C2C12 myotubes are a useful model to analyze GLUT4 translocation in skeletal muscle [23]. Akt (PKB) stimulates glucose uptake via modulation of GLUT4 [24]. TT treatment increased the expression levels of membrane GLUT4 (at 1, 5, 10, and 25 μg/mL) and phospho-Akt/total-Akt (at 10 and 25 μg/mL). TT stimulated glucose transport partially via an insulin-dependent pathway *in vitro*. TT1-, TT2-, TT3- Feno-, or Metf-treated mice exhibited 1.40-, 1.98-, 2.59-, 1.95-, or 1.91-fold increases in the expression levels of muscular membrane GLUT4 compared to the vehicle-treated HF group. These results suggest that TT-treated mice exhibited significantly increased muscular glucose uptake and improved insulin sensitivity to exert an antidiabetic activity.

This study also measured the expression levels of phospho-AMPK following TT treatment. AMPK phosphorylation increases lipid and glucose catabolism [4]. During medication metformin, the pathway of AMPK phosphorylation belongs to the different superior modulator of GLUT4 translocation [22]. We observed that TT treatment significantly increased the expression levels of muscular and hepatic phospho-AMPK/total AMPK. Metformin is used clinically in the management of diabetes, and it enhances glucose uptake in skeletal muscle and inhibits hepatic glucose production [4,25]. These data indicated that TT treatment exhibited resistance to HFD-induced hyperglycemia via enhancing AMPK activation and increasing GLUT4 protein content.

Another potential mechanism of TT-mediated insulin sensitivity was investigated. The enzyme 11β hydroxysteroid dehydrogenase (11β-HSD1) converts dehydrocorticosterone to corticosterone, and 11β-HSD1 knockout mice exhibit resistance to HFD-induced insulin resistance [26]. Treatment with TT, Feno, and Metf reduced the mRNA levels of hepatic 11β-HSD1, which suggests that this enzyme also contributed to the amelioration of insulin resistance. Glucose-6-phosphatase (G6 Pase) plays a central role in gluconeogenesis [27]. Metformin improves glycemic control primarily via suppression of hepatic glucose production and enhancement of peripheral glucose uptake [4]. Our results demonstrated that TT-treated mice exhibited increased expression levels of hepatic phospho-AMPK/total AMPK but decreased mRNA levels of G6 Pase. Collectively, these results demonstrated that TT stimulated glucose uptake and enhanced muscular and hepatic AMPK activation. This inhibition of G6 Pase mRNA levels decreased hepatic glucose production. These results support the effects of TT in the improvement of glycemic control via enhancement of AMPK activation and muscular membrane GLUT4 protein contents and decreasing hepatic gluconeogenesis.

This study also investigated the antihyperlipidemic effects and mechanisms of TT. The triglyceride-lowering effect of TT (19.4%−21.8%) in the animal study was comparable to Feno, which is a PPARα agonist with triglyceride-lowering effects [20,21]. Evidence suggests that the PPARα receptor is abundantly expressed in liver, and it controls fatty acid oxidation [21]. PPARα ligands are used clinically to lower plasma triglyceride levels in patients with dyslipidemia [20,21]. FAS is the core mediator in the synthesis of fatty acids [28]. TT-treated mice exhibited increased hepatic and adipose expression levels of PPAR α but decreased FAS expressions. These results suggest that TT, Feno, and Metf had reduced blood triglyceride levels by inhibiting hepatic FAS expression and enhancing PPARα. These results also suggest that TT may exhibit therapeutic potential in the management of type 2 diabetes accompanied with hypertriglyceridemia.

This study also examined TT-mediated suppression of lipogenesis, including lipogenic enzymes aP2 and GPAT. Evidence suggests that mice with aP2 deficiency are protected from the development of dyslipidemia, hyperglycemia, insulin resistance, and fatty liver disease in genetic and dietary obesity [29]. Ablation of aP2 enhanced liver accumulation of longer-chain fatty acids, which reduced SREBP1c expression and several downstream lipogenic enzymes [29]. SREBP1c may up-regulate a variety of lipogenic genes [30]. PPARα-deficient mice exhibit a dysregulation of SREBP-regulated genes [21]. GPAT catalyzes the initial step of glycerolipid synthesis, and it plays a key role in the regulation of triacylglycerol (TAG) synthesis. The mRNA levels of GPAT are highly expressed in the liver, but low protein expression and changes in the modulation of mitochondrial GPAT would result in disorders of lipid metabolism, such as type 2 diabetes [31]. TT-treated mice exhibited protecting against HFD-induced insulin resistance and ameliorated hepatic steatosis, which may be partially due to a decrease in hepatic mRNA levels of aP2, SREBP1c, and GPAT, reduced cellular TAG synthesis and reduced blood TG.

Carnitine palmitoyl transferase I (CPT-1) is the rate-limiting enzyme for entrance to mitochondria fatty acids oxidation [32]. An increased hepatic expression of uncoupling protein 3 (UCP3) was observed in states of enhanced hepatic fatty acid catabolism, including a high-fat diet plus treatment with fenofibrate or fenofibrate alone [31,33]. UCP3 expression is involved in hepatic metabolism, including increases in fatty acid-mediated mitochondrial oxidation, and it is associated with lipid catabolism [34,35]. TT treatment enhanced hepatic mRNA levels of CPT-1a and UCP3 (significantly in TT2- and TT3-treated mice) and increased expression levels of PPARα in our animal study. These effects may be involved in increased fatty acid oxidation.

The combination of enhanced fatty acid oxidation and decreased TAG synthesis by TT, Metf, and Feno may contribute to a decrease in hepatic fat storage and induce hepatic triglyceride output, which led to a decrease in circulating TG and hepatic steatosis.

There is an inverse relationship between plasma leptin or mRNA expression of leptin, and insulin sensitivity [36]. Our results demonstrated that leptin concentration was increased in HFD-fed mice, which is consistent with a prior study [37]. TT administration reduced leptin levels, which suggests that TT provides a beneficial therapeutic advantage in the regulation of adipocytes to improve insulin sensitivity. Leptin stimulated fatty-acid oxidation via activation of an AMP-activated protein [38]. Therefore, TT could directly activate AMPK or decrease leptin levels via the induction of AMPK activation. 

PPARγ is highly expressed in adipocytes [39]. PPARγ is the master regulator of adipocyte differentiation and lipid accumulation [40]. TT, Feno, and Metf administration reduced the expression of lipogenic PPARγ and FAS. These reductions led to inhibited adipocyte differentiation and reduced lipid accumulation. Lipid is generally stored in adipose tissue, and blood TG levels fluctuate. The liver is the major organ of lipid metabolism. TT likely removed fat from adipose tissue to peripheral tissues and increased lipid catabolism via reduced hepatic lipogenic FAS expression and enhanced fatty acid oxidation via PPARα. TT decreased the adipocyte adipogenesis gene PPARγ and lipogenic FAS. All of these changes led to reduced TG levels in the liver, blood, and adipose tissue. Histology analyses revealed that TT treatment decreased adipocyte size and almost abolished hepatic lipid droplets.

TT exhibited a total cholesterol-lowering effect (21.1%−26.4%) that was comparable to the Feno-treated group (24.5%). TT-treated mice exhibited reduced mRNA levels of SREBP2, which is associated with total cholesterol synthesis [41]. The demonstrated reduction of blood cholesterol by TT was caused by the inhibition of total cholesterol synthesis.

## 4. Materials and Methods

### 4.1. Chemicals

Antibodies to GLUT4 (no. sc-79838) were obtained from Santa Cruz Biotech (Santa Cruz, CA, USA). Phospho-AMPK (Thr^172^), PPARα (no. ab8934), and PPARγ (no. ab45036) antibodies were purchased from Abcam Inc. (Cambridge, MA, USA). FAS (no. 3180), phospho-Akt (Ser^473^) (no. 4060), total AMPK (Thr^172^), and β-actin (no. 4970) antibodies were obtained from Cell Signaling Technology (Danvers, MA, USA). Secondary anti-rabbit antibodies were purchased from Jackson ImmunoRes. Lab., Inc. (West Grove, PA, USA).

### 4.2. The Active Compound Determination

The mycelium of *Antrodia camphorata* (=*Antrodia cinnamomea*) was obtained from the Konald Biotech Co. Ltd. (Chiayi City, Taiwan). Freeze-dried powders of the mycelia of *Antrodia camphorata* (3.0 kg) were extracted three times with methanol (12 L) at room temperature (4 days × 3). The methanol extract was evaporated in vacuo to yield a brown residue, which was suspended in H_2_O (1 L) and partitioned with ethyl acetate (1 L × 3). The EtOAc fraction (200 g) was chromatographed on silica gel using mixtures of hexane and EtOAc of increasing polarity as eluents and further purified with HPLC (Shimadzu CL 20-A, Kyoto, Japan) on a Hibar pre-packed column RT 250-10 with chloroform: ethyl acetate (7:1). The flow rate was 3 mL/min, and the injection volumes of samples were 100 μL. This part of the procedure was performed in accordance with prior reports [8]. Dehydroeburicoic acid (TT) was isolated by HPLC on a Hibar pre-packed column RT 250-10 with chloroform: ethyl acetate (7:1) and a refractive index (RI) (Knauer RI detector 2400). The flow rate was 3 mL/min, and the injection volumes of samples were 100 μL [10,12]. The obtained yields of TT were approximately 0.2% (*w*/*w*), and the purity was over 99% [8,10].

The fruiting body of *A. camphorata* was purchased from the Balay Biotechnology Corporation in Hsinchu City, Taiwan. A voucher specimen (CMPC393) was deposited and identified by China Medical University. The fruiting bodies of AC (3.0 kg) were extracted three times with methanol followed by chromatography using 50% ethyl acetate and 50% hexane. This procedure was performed as previously reported [42]. The purity of AnK was over 99%. The analytical instruments were an HPLC (Shimadzu CL 20-A, Kyoto, Japan); the HPLC Column, TOSOH TSKgel DS-80Ts, and the analytical condition was 100% MeOH.

### 4.3. Cell Culture

C2C12 skeletal myoblasts (ATCC, CRL-1772) were maintained in growth media consisting of Dulbecco’s modified Eagle’s medium (DMEM) (Gibco BRL, Bedford, MA, USA) and supplemented with 10% Fetal Bovine Serum (FBS) (Hyclone, South Logan, UT, USA) and 100 U/mL penicillin/100 µg/mL streptomycin (Gibco BRL), and split 1:4 using 0.05% trypsin when 80% confluent. Myoblasts were diluted and placed in a 9 cm dish. Cells were cultured to achieve 80%–90% confluency and changed growth media as 2% FBS/DMEM every 24 h for 5–7 days and cultured as previously reported [10].

### 4.4. Detection of Expression Levels of Membrane GLUT4 and Phosphorylation of Akt (Ser^473^) in Vitro

The procedure was performed as previously reported [8,43,44]. Differentiated C2C12 cells were serum-starved in DMEM/BSA (2 h at 37 °C) prior to incubation with MeOH crude extract (at 20, 100, 200, and 500 μg/mL), test compounds (at 1, 5, 10, and 25 μg/mL) or vehicle for 25 min or 100 nM insulin for 25 min, as previously described [45]. The homogenates were centrifuged, and the pellet was resuspended. Assays were performed within the membrane. Protein concentrations were analyzed using a BCA assay (Pierce, Rockford, IL, USA), and equal amounts of protein were diluted four times in SDS sample buffer, and subjected to SDS PAGE. Proteins were detected using Western blotting with antibodies specific for Akt, phospho-Akt Ser^473^, and GLUT4, and the analyses of band density were performed as previously reported [10].

### 4.5. Animal Study

Animal studies were performed and approved under the guidelines of the Affidavit of Approval of Animal Care and Use Protocol Central Taiwan University of Science and Technology (No: 103-CTUST-09) (10 July 2015). We used 4-week old male C57BL/6J mice (*n* = 63) obtained from the National Laboratory Animal Breeding Center. A group of control mice (CON) received control diet (CD; Diet 12450B, Research Diets, Inc., New Brunswick, NJ, USA; low-fat diet) (*n* = 9) for 12 weeks. The other mice (*n* = 54) received a high-fat diet (HFD) (Diet 12451, Research Diets, Inc.) over 12 weeks [7,46]. The control diet or HFD contained10% fat or 45% fat, respectively. The HFD mice were randomly divided into six groups (*n* = 9, each set) followed by an eight-week CD- or HFD-induction. Three groups were treated with dehydroeburicoic acid (TT) at 10, 20, or 40 mg/kg/day body weight (groups TT1, TT2, and TT3, respectively). Three comparative groups were administered fenofibrate (Feno: 0.25 g/kg/day body weight, Sigma Chemical Co., St Louis, MO, USA), metformin (Metf: 0.3 g/kg/day body weight, Sigma Chemical Co., St Louis, MO, USA) or distilled water. All treatments were administered via oral gavage once daily for 28 days. The high-fat control (HF) group was treated with similar volumes of distilled water. All mice were fasted overnight, and blood was collected from the retro-orbital sinus under ether anesthesia. At the end of the experiment, the mice were sacrificed via carbon dioxide inhalation. Liver, adipose tissue, and skeletal muscle were removed and immediately stored at −80 °C until use. Plasma samples were collected via centrifugation of whole blood at 1600× *g* for 15 min at 4 °C, followed by plasma separation within 30 min. Aliquots of plasma were obtained for triglyceride (TG) and total cholesterol analysis. The metabolic parameters, including body weight, weight gain, and food intake, were performed as the followings. Body weight was daily measured at the same time throughout the study. Body weight gain is considered as the difference between one day and the next day. The amount of pellet food was weighed, and followed by weighing the amount of remaining food after 24 h. The difference is represented the daily food intake.

### 4.6. Measurements of Blood Glucose Levels and Biochemical Parameters

Blood samples were collected from the retro-orbital sinuses of 12 h fasted mice. Blood glucose levels were determined by the glucose oxidase method (Model 1500; Sidekick Glucose Analyzer; YSI Incorporated, Yellow Springs, OH, USA). The levels of TG, TC, and free fatty acids were determined using commercial assay kits in accordance with manufacturer’s directions (Triglycerides-*E* test, Cholesterol-*E* test and FFA-C test, Wako Pure Chemical, Osaka, Japan). Insulin and leptin levels were measured by enzyme-linked immunosorbent assay (ELISA) (mouse insulin ELISA kit, Mercodia, Uppsala, Sweden; mouse leptin ELISA kit, Morinaga, Yokohama, Japan).

### 4.7. Histopathology Examination

Parts of visceral adipose and liver specimens were fixed with formalin (200 g/kg) neutral buffered solution and embedded in paraffin. Sections (8 μm) were cut and stained with hematoxylin and eosin. Images were photographed by a microscope (Olympus BX51, Olympus, Tokyo, Japan).

### 4.8. Liver Lipids Analysis

Liver lipids were analyzed in accordance with previous procedures [47]. For the hepatic lipid extraction, the liver samples (0.375 g) were homogenized with distill water (1 mL) for 5 min. Finally, the dried pellet was resuspended in ethanol (0.5 mL) and analyzed using a triglycerides kit as blood triglycerides kit.

### 4.9. Relative Quantization of mRNA and Western Blotting

Relative mRNA quantification (primers are described in Table 2) and immunoblots for the measurement of muscular membrane GLUT4 and hepatic and muscular phospho-AMPK (Thr^172^) proteins were performed as described in previous studies [9,10,46]. Liver tissue was analyzed for PPARα and FAS protein content. Adipose tissue was analyzed for PPARγ and FAS protein content. Skeletal muscle was analyzed for GLUT4 expression. The total membrane fraction was measured, and the expression levels of GLUT4, phospho-AMPK, and total AMPK were determined using Western blotting as previously described [9,10,46].

### 4.10. Statistics

Results are presented as the means + standard errors. Comparisons between groups were performed using non-parametric with Kruskal-Wallis H test, and followed by Mann-Whitney *U* test. All *p-*values less than 0.05 were considered significant.

## 5. Conclusions

**Figure 7:**
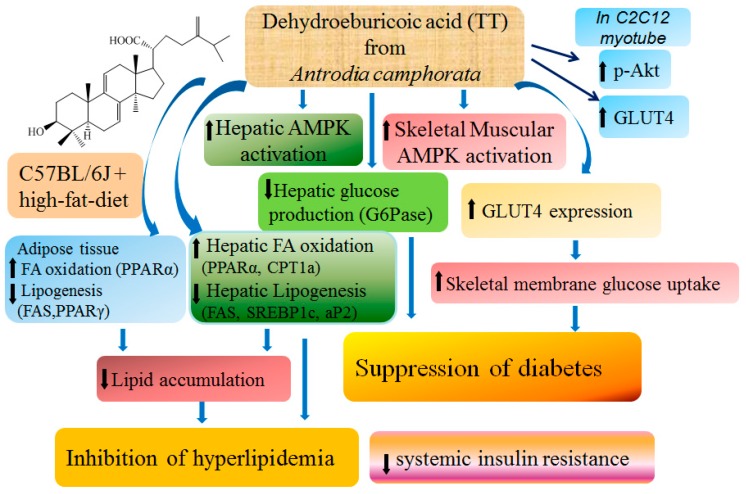
The graphic abstract of dehydroeburicoic acid (TT). ↑: increase; ↓: decrease.

In summary (Figure 7), treatment with insulin and the methanol crude extract (CruE), insulin, AnK, and TT increased the expression levels of membrane GLUT4 and phospho-Akt (Ser^473^)/total Akt at different concentrations *in vitro.* This shows that TT stimulates glucose transport partially via an insulin-dependent pathway *in vitro*. TT treatment in mice not only lowered blood glucose and insulin levels, but also reduced triglycerides and total cholesterol levels; and finally ameliorated insulin resistance. TT exhibited antidiabetic effects by markedly increasing the expression levels of skeletal muscle membrane GLUT4 and decreasing mRNA levels of G6 Pase, which lowered blood glucose levels. TT and Metf treatment in mice also increased hepatic and skeletal muscle AMPK activation. TT treatment increased the expression levels of fatty acid oxidation genes, including PPARα, and the mRNA levels of CPT1a and decreased the expression of lipogenic FAS and PPARγ and hepatic mRNA levels of SREBP1c, aP2, and GPAT, with the net effect of lowering blood triglycerides, hepatic steatosis, and total cholesterol levels. These results support the effects of TT and Metf in the improvement of glycemic control via enhancement of AMPK activation and muscular membrane GLUT4 protein contents. TT activates AMPK or Akt phosphorylation to increase GLUT4 translocation in muscles or *in myotubes*, which lead to a reduction in systemic insulin resistance, fat accumulation in adipose and liver tissue. We observed that TT exhibited excellent antidiabetic activity in HFD-fed mice. Our results suggest that TT exhibits excellent beneficial potential for the treatment of type 2 diabetes associated with hypertriglyceridemia and hypercholesterolemia.

## Figures and Tables

**Table 1:** Effects of dehydroeburicoic acid (TT) on absolute tissue weight (mg), food intake, and blood parameters in HFD-fed mice. All values are means ± SE (*n* = 9). ^a^ *p* < 0.05, ^b^ *p* < 0.01, and ^c^ *p* < 0.001 compared with the control (CON) group; ^d^ *p* <0.05, ^e^ *p* <0.01, and ^f^ *p* < 0.001 compared with the high-fat plus vehicle (distilled water) (HF) group. TT1, TT2, and TT3: dehydroeburicoic acid (TT1: 10, TT2: 20, and TT3: 40 mg/kg bodyweight); Feno: fenofibrate (250 mg/kg body weight); Metf: metformin (300 mg/kg body weight). EAWT, epididymal white adipose tissue; RWAT, retroperioneal white adipose tissue; MWAT, mesenteric white adipose tissue; Visceral fat represented epididymal WAT plus retroperitoneal WAT; FFA, plasm free fatty acid; BAT, brown adipose tissue.

Parameter	CON	HF	HF + TT1	HF + TT2	HF + TT3	HF + Feno	HF + Metf
Dose (mg/kg/day)	–	–	10	20	40	250	300
Absolute tissue weight (mg)							
EWAT	423.6 ± 22.4	963.1 ± 68.7 ^c^	798.9 ± 43.2 ^c^	784.3 ± 102.0 ^b^	667.8 ± 53.2 ^c,e^	663.1 ±47.0 ^b,e^	482.1 ± 31.5 ^f^
MWAT	246.3 ± 7.3	369.5 ± 24.2 ^b^	361.3 ± 41.1 ^a^	340.9 ± 28.4	304.6 ± 29.5	275.9 ± 26.1 ^d^	198.2 ± 24.2 ^f^
RWAT	133.5 ± 11.8	470.4 ± 45.6 ^c^	343.9 ± 23.4 ^c^	331.9 ± 47.5 ^c^	311.4 ± 33.6 ^c,e^	268.6 ± 26.2 ^b,e^	180.4 ± 15.7 ^f^
Visceral fat	557.0 ± 31.5	1433.4 ± 112.9 ^c^	1142.9 ± 32.4 ^b^	1116.2 ± 131.5 ^c,d^	979.3 ± 86.2 ^c,e^	931.7 ± 70.6 ^b,e^	662.4 ± 44.0 ^f^
Liver	878.5 ± 28.0	889.0 ± 15.3	848.3 ± 30.6 ^d^	841.8 ± 13.4 ^a,d^	802.8 ± 24.4 ^d^	882.4 ± 24.1	1959.0 ± 105.6 ^c,f^
Spleen	76.0 ± 2.6	86.1 ± 1.7	77.7 ± 3.0	81.5 ± 10.1	82.3 ± 3.7	74.6 ± 4.5	81.0 ± 3.5
BAT	135.6 ± 9.7	249.0 ± 15.3 ^c^	228.6 ± 23.9 ^c^	197.3 ± 12.9 ^c,a^	198.1 ± 14.2 ^c,a^	170.4 ± 11.0 ^a,e^	134.2 ± 5.1 ^f^
Weight gain (g)	1.56 ± 0.19	2.89 ± 0.27 ^c^	2.24 ± 0.14 ^a,d^	0.89 ± 0.26 ^f^	0.83 ± 0.21 ^a,f^	0.46 ± 0.24 ^b,f^	0.52 ± 0.29 ^a,f^
Final body weight (g)	24.72 ± 0.48	28.55 ± 0.64 ^c^	27.52 ± 0.67 ^b^	26.47 ± 0.57 ^d^	26.53 ± 0.91 ^d^	26.04 ± 0.60 ^d^	26.35 ± 0.85 ^d^
Food intake (g/day/mouse)	2.45 ± 0.05	2.26 ± 0.06 ^a^	2.21 ± 0.05 ^b^	2.16 ± 0.05 ^c^	2.07 ± 0.04 ^c,d^	2.05 ± 0.06 ^c,d^	2.25 ± 0.06 ^a^
Liver lipids							
total lipid (mg/g)	56.9 ± 1.4	89.7 ± 2.1 ^c^	64.4 ± 1.8 ^b,f^	61.7 ± 1.9 ^f^	57.9 ± 1.4 ^f^	62.0 ± 2.1 ^f^	61.1 ± 1.9 ^f^
triacylglycerol (μmol/g)	45.9 ± 3.0	78.4 ± 4.6 ^c^	53.0 ± 2.6 ^f^	48.0 ± 2.4 ^f^	47.1± 2.0 ^f^	48.6 ± 2.0 ^f^	49.0 ± 2.1 ^f^
Blood profiles							
FFA (meq/L)	0.96 ± 0.13	1.28 ± 0.19 ^c^	0.95 ± 0.09 ^f^	0.87 ± 0.120 ^f^	0.81 ± 0.06 ^b,f^	0.86 ± 0.08 ^a,f^	0.85 ± 0.09 ^a,f^
Blood glucose (mg/dL)	78.56 ± 1.71	137.44 ± 2.62 ^c^	90.44 ± 1.80 ^c,f^	82.89 ±1.91 ^f^	77.78 ± 2.44 ^f^	86.56 ± 3.23 ^a,f^	87.33 ± 2.78 ^a,f^
TG (mg/dL)	83.82 ± 2.14	105.27 ± 1.10 ^c^	84.85 ± 1.36 ^f^	83.63 ± 2.42 ^f^	82.31 ± 1.60 ^f^	83.35 ± 2.09 ^f^	82.07 ± 1.53 ^f^
TC (mg/dL)	101.70 ± 1.17	152.71 ± 4.15 ^c^	120.55 ± 0.92 ^c,f^	114.93 ±2.74 ^c,f^	112.46 ±1.76 ^c,f^	115.25 ± 3.05 ^c,f^	117.20 ± 3.16 ^c,f^
Insulin (μg/L)	2.247 ± 0.010	3.563 ± 0.004 ^c^	2.982 ± 0.024 ^b,f^	2.625 ± 0.017 ^b,f^	2.190 ± 0.025 ^f^	2.402 ± 0.029 ^b,f^	2.467 ± 0.025 ^b,f^
Leptin(ng/mL)	6.640 ± 0.168	14.116 ± 0.244 ^c^	9.827 ± 0.052 ^c,f^	8.468 ± 0.182 ^c,f^	6.439 ± 0.091 ^f^	7.507 ± 0.144 ^b,f^	7.359 ± 0.1325 ^b,f^

**Table 2:** Primers used in this study.

Gene	Accession Number	Forward Primer and Reverse Primer	PCR Product (bp)	Annealing Temperature (°C)
Liver				
*G6Pase*	NM_008061.3	F: GAACAACTAAAGCCTCTGAAAC	350	50
		R: TTGCTCGATACATAAAACACTC		
*SREBP1c*	NM_011480	F: GGCTGTTGTCTACCATAAGC	219	48
		R: AGGAAGAAACGTGTCAAGAA		
*GPAT*	BC019201.1	F: CAGTCCTGAATAAGAGGT	441	51
		R: TGGACAAAGATGGCAGCAGA		
*apo C-III*	NM_023114.3	F: CAGTTTTATCCCTAGAAGCA	349	47
		R: TCTCACGACTCAATAGCTG		
*CPT1a*	BC054791.1	F: CTTGTGACCCTACTACATCC	332	51
		R: TCATAGCAGAACCTTAATCC		
*SREBP2*	AF289715.2	F: ATATCATTGAAAAGCGCTAC	256	48
		R: ATTTTCAAGTCCACATCACT		
*aP2*	NM_024406	F: TCACCTGGAAGACAGCTCCT	142	52
		R: TGCCTGCCACTTTCCTTGT		
*UCP3*	NM_009464	F: GAGGTGACTACAGCCTTCTG	242	51
		R: TAGGAAGTGCTTCCATGTCT		
*11β-HSD1*	NM_008288.2	F: AAGCAGAGCAATGGCAGCAT	300	50
		R: GAGCAATCATAGGCTGGGTCA		
*β-actin*	NM_007392	F: TCTCCACCTTCCAGCAGATGT	92	60
		R: AGCTCAGTAACAGTCCGCCTAGA		

## Abbreviations

AMPK	AMP-activated protein kinase
aP2	adipocyte fatty acid binding protein 2
11β-HSD1	11β hydroxysteroid dehydrogenase
BAT	brown adipose tissue
CON	control
CPT1a	carnitine palmitoyl transferase Ia
EWAT	epididymal white adipose tissue
FAS	fatty acid synthase
Feno	fenofibrate
FFA	free fatty acid
GAPDH	glyceraldehyde-3-phosphate dehydrogenase
G6 Pase	glucose-6-phosphatase
GPAT	glycerol-3-phosphate acyltransferase
GLUT4	glucose transporter 4
HFD	high-fat-diet
Metf	metformin
MWAT	mesenteric white adipose tissue
PEPCK	phosphoenolpyruvate carboxykinase
phospho-AMPK	AMPK phosphorylation
PPAR	peroxisome proliferator-activated receptor
RT-PCR	reverse transcription-polymerase chain reaction
RWAT	retroperitoneal white adipose tissue
SREBP	sterol regulatory element binding protein
TC	total cholesterol
TG	triglyceride
UCP3	uncoupling protein 3
WAT	white adipose tissue
